# CYP2S1 is a synthetic lethal target in BRAF^V600E^-driven thyroid cancers

**DOI:** 10.1038/s41392-020-00231-6

**Published:** 2020-09-11

**Authors:** Yiqi Li, Xi Su, Chao Feng, Siyu Liu, Haixia Guan, Yue Sun, Nongyue He, Meiju Ji, Peng Hou

**Affiliations:** 1 Department of Endocrinology, The First Affiliated Hospital of Xi’an Jiaotong University, Xi’an, 710061 P.R. China; 2 Department of Endocrinology and Metabolism, The First Affiliated Hospital of China Medical University, Shenyang, 110001 P.R. China; 3 Philips Institute for Oral Health Research, Virginia Commonwealth University, Richmond, VA 23298 USA; 4 State Key Laboratory of Bioelectronics, Southeast University, Nanjing, 210096 P.R. China; 5 Center for Translational Medicine, The First Affiliated Hospital of Xi’an Jiaotong University, Xi’an, 710061 P.R. China; 6 Key Laboratory for Tumor Precision Medicine of Shaanxi Province, The First Affiliated Hospital of Xi’an Jiaotong University, Xi’an, 710061 P.R. China

**Keywords:** Tumour biomarkers, Cancer therapy

## Abstract

BRAF^V600E^ is the most common genetic alteration and has become a major therapeutic target in thyroid cancers; however, intrinsic feedback mechanism limited clinical use of BRAF^V600E^ specific inhibitors. Synthetic lethal is a kind of interaction between two genes, where only simultaneously perturbing both of the genes can lead to lethality. Here, we identified CYP2S1 as a synthetic lethal partner of BRAF^V600E^ in thyroid cancers. First, we found that CYP2S1 was highly expressed in papillary thyroid cancers (PTCs) compared to normal thyroid tissues, particularly in conventional PTCs (CPTCs) and tall-cell PTCs (TCPTCs), and its expression was positively associated with BRAF^V600E^ mutation. CYP2S1 knockdown selectively inhibited cell proliferation, migration, invasion and tumorigenic potential in nude mice, and promoted cell apoptosis in BRAF^V600E^ mutated thyroid cancer cells, but not in BRAF wild-type ones. Mechanistically, BRAF^V600E^-mediated MAPK/ERK cascade upregulated CYP2S1 expression by an AHR-dependent pathway, while CYP2S1 in turn enhanced transcriptional activity of AHR through its metabolites. This AHR/CYP2S1 feedback loop strongly amplified oncogenic role of BRAF^V600E^ in thyroid cancer cells, thereby causing synthetic lethal interaction between CYP2S1 and BRAF^V600E^. Finally, we demonstrated CYP2S1 as a potential therapeutic target in both BRAF^V600E^-drived xenograft and transgenic mouse models by targetedly delivering CYP2S1-specific siRNA. Altogether, our data demonstrate CYP2S1 as a synthetic lethal partner of BRAF^V600E^ in thyroid cancers, and indicate that targeting CYP2S1 will provide a new therapeutic strategy for BRAF^V600E^ mutated thyroid cancers.

## Introduction

The incidence rate of thyroid cancer increased rapidly all over the world since the 1970s, until now it has become the most common endocrine malignancy.^1^ Mitogen-activated protein kinase/extracellular signal-regulated protein kinase (MAPK/ERK) signaling is highly selected in thyroid cancers, particularly in papillary thyroid cancers (PTCs). Among MAPK/ERK pathway-related genetic alterations, BRAF^V600E^ mutation is the most common in PTCs.^2^ However, unlike melanoma, which is known as another kind of BRAF^V600E^ mutation dominated malignancy, patients diagnosed with BRAF^V600E^-mutated thyroid cancers seldom benefit from BRAF^V600E^ specific small-molecule inhibitors because of the existence of HER3 feedback activation.^3^ Thus, it is pressing to develop an effective therapeutic strategy for this type of thyroid cancer.

Synthetic lethal is a kind of interaction between two genes, where only simultaneously perturbing both of the genes can lead to lethality. This will provide an alternative paradigm to target “undruggable but important” targets in human cancers.^4^ Given that metabolic alterations act as a hallmark of cancer, thus approaches utilize metabolic properties specific in distinct oncogenic backgrounds can be considered to achieve synthetic lethality in cancer cells.^5,6^ A previous study has identified eight potential metabolic-related synthetic lethality partners of BRAF^V600E^ in melanoma through a high-throughput screening.^7^ We thus analyzed the expression of these genes in PTCs using The Cancer Genome Atlas (TCGA) dataset, and found increased expression of CYP2S1, an orphan cytochrome P450 (CYP) enzyme,^8^ in PTCs compared to control subjects. In addition, its expression was strongly associated with BRAF^V600E^ mutation, suggesting that CYP2S1 may be selectively essential in BRAF^V600E^ mutated thyroid cancers.

The CYP superfamily includes the genes that code phase I enzymes metabolism both endogenous and exogenous substrates.^9^ There is evidence reporting that dioxin can induce CYP2S1 expression by an aryl hydrocarbon receptor (AHR)-dependent pathway, and CYP2S1 may be involved in metabolic activation of carcinogens which bring about harmful effects.^10,11^ The ability of CYP2S1 to metabolism endogenous substrates is still controversial, and current evidence suggests that CYP2S1 is capable of metabolize cyclooxygenase-derived and lipoxygenase-derived eicosanoids.^12^ Considering that the eicosanoids are “double edged sword” for carcinogenesis^13^ and increased expression of CYP2S1 in PTCs, thus there is a high possibility that CYP2S1 participates in malignant phenotypes of thyroid cancer cells, particularly in BRAF^V600E^ mutated ones.

In this study, we identify CYP2S1 as a synthetic lethal partner of BRAF^V600E^ in thyroid cancer, and preliminarily reveal the mechanism underlying a synthetic lethal interaction between CYP2S1 and BRAF^V600E^. This study will provide a potential therapeutic strategy for BRAF^V600E^-mutated thyroid cancers.

## Results

### CYP2S1 is identified as a potential synthetic lethal partner of BRAF^V600E^ in thyroid cancer cells

**Fig. 1:**
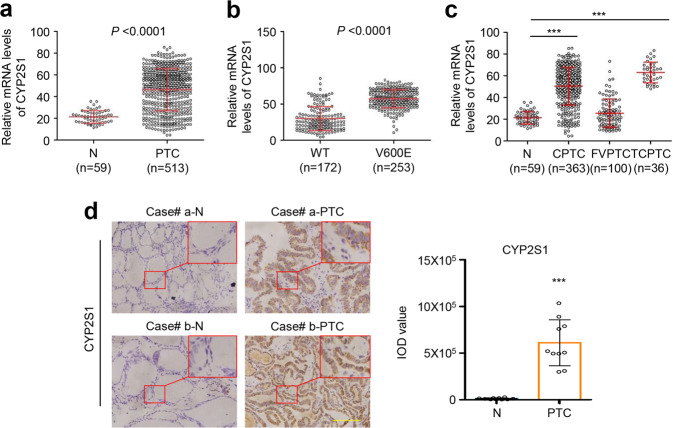
Increased expression of CYP2S1 in PTCs. **a** High expression of CYP2S1 in PTCs compared to normal thyroid tissues (N) from TCGA database. **b** High expression of CYP2S1 in BRAF^V600E^ mutated PTCs (V600E) compared to BRAF wild-type ones (WT). **c** CYP2S1 expression in different histological subtypes of PTCs. CPTC conventional PTC, FVPTC follicular variants PTC, TCPTC tall-cell PTC. **d** Evaluation of CYP2S1 expression in ten PTCs and their matched noncancerous tissues (N) by IHC staining. Representative IHC images were shown in left panel. Quantitative illustration of CYP2S1 proteins was shown in right panel. Error bars represent SD. ***P < 0.001. Scale bars, 200 µm

Eight metabolism related genes including HMGCL, HMGCS1, CYP39A1, CYP2C9, CYP2E1, CYP2J2, CYP2S1, and CSGLCA-T have been screened to be potential synthetic lethal partners of BRAF^V600E^ in melanomas, and HMGCL was finally validated as a synthetic lethal target of BRAF^V600E.^^7^ Similar to melanomas, there is a high prevalence of BRAF^V600E^ mutation in thyroid cancers.^14,15^ Thus, we first analyzed the expression of these eight genes in PTCs from TCGA database. The results showed that the expression of HMGCL, CYP2J2, CYP2S1 and CSGLCA-T was significantly elevated in PTCs, especially the latter three genes (Fig. 1a, Supplementary Fig. 1) compared to control subjects. Of them, the expression of CYP2S1 and CSGLCA-T was positively associated with BRAF^V600E^ mutation, particularly the former (Fig. 1b, Supplementary Fig. 2). Considering that BRAF^V600E^ mutation is also found in other types of cancer such as melanomas, colon cancers, and lung adenocarcinomas,^16^ we also investigated the association of CYP2S1 expression with BRAF^V600E^ mutation in the above cancers. As shown in Supplementary Fig. 3, CYP2S1 expression was not clearly associated with BRAF^V600E^ mutation in melanomas and lung adenocarcinomas. Moreover, unlike thyroid cancer, CYP2S1 expression was downregulated in BRAF-mutated colon adenocarcinomas compared to BRAF wild-type ones. Taken together, we speculate that CYP2S1 may be a potential synthetic lethal partner of BRAF^V600E^ in thyroid cancers.

In general, PTCs are classified into conventional PTC (CPTC), follicular variants PTC (FVPTC), tall-cell PTC (TCPTC), and other rare variants based on their distinct pathological features, growth patterns and behaviors.^17^ TCPTC is more aggressive and the prevalence of BRAF^V600E^ mutation is ~100%, while its prevalence in FVPTCs is relatively low.^2,18^ This was supported by the data from TCGA dataset that CYP2S1 expression was much higher in TCPTCs than FVPTCs and normal controls (Fig. 1c). In addition, using IHC assay, we confirmed that CYP2S1 expression was dramatically increased in PTCs compared to their paired noncancerous tissues (Fig. 1d).

**Fig. 2:**
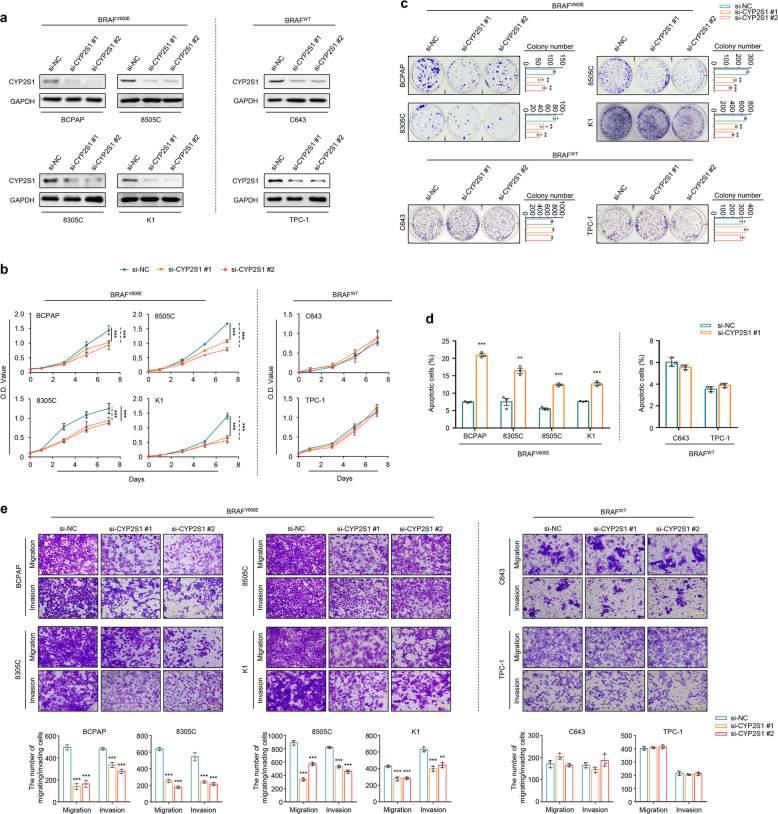
Identification of CYP2S1 as a synthetic lethal partner of BRAF^V600E^ in thyroid cancer cells. **a** Western blot analysis was performed to validate inhibition of CYP2S1 expression by two different siRNAs (si-CYP2S1 #1 and si-CYP2S1 #2) in BCPAP, 8305C, 8505C, and K1 cells harboring *BRAF*^*V600E*^ mutation (BRAF^V600E^) and C643 and TPC-1 cells harboring wild-type BRAF (BRAF^WT^). GAPDH was used as a loading control. **b** The effect of CYP2S1 knockdown on cell proliferation in the indicated cell lines was monitored by MTT assay. **c** The effect of CYP2S1 knockdown on colony formation in the indicated cell lines. Representative images were shown in left panels. Quantitative analysis of colony numbers was shown in right panels. **d** Flow cytometry analysis was performed to evaluate the effect of CYP2S1 knockdown on cell apoptosis in the indicated cell lines. **e** Transwell assays were performed to evaluate the effect of CYP2S1 knockdown on cell migration and invasion. Error bars represent SD. Data were from three independent experiments. **P < 0.01; ***P < 0.001

To determine whether CYP2S1 serves as a synthetic lethal partner of BRAF^V600E^ in thyroid cancers, we first knocked down CYP2S1 expression in four BRAF^V600E^-mutated thyroid cancer lines (BCPAP, 8305C, 8505C, and K1) and two BRAF wild-type thyroid cancer cell lines (C643 and TPC-1), as shown in Fig. 2a. The results showed that CYP2S1 knockdown significantly inhibited cell proliferation in BRAF^V600E^-mutated thyroid cancer cell lines, but not in BRAF wild-type ones (Fig. 2b), also supported by colony formation assays (Fig. 2c). Next, we tested the effect of CYP2S1 depletion on cell apoptosis. As expected, CYP2S1 knockdown strongly induced cell apoptosis in BRAF^V600E^-mutated thyroid cancer cell lines, but not in BRAF wild-type ones (Fig. 2d). Besides, our data also showed that CYP2S1 knockdown selectively decreased migration and invasion ability of BRAF^V600E^-mutated thyroid cancer cells compared to the control (Fig. 2e).

### Validation of CYP2S1 as a synthetic lethal partner of BRAF^V600E^ in xenograft tumor models

**Fig. 3:**
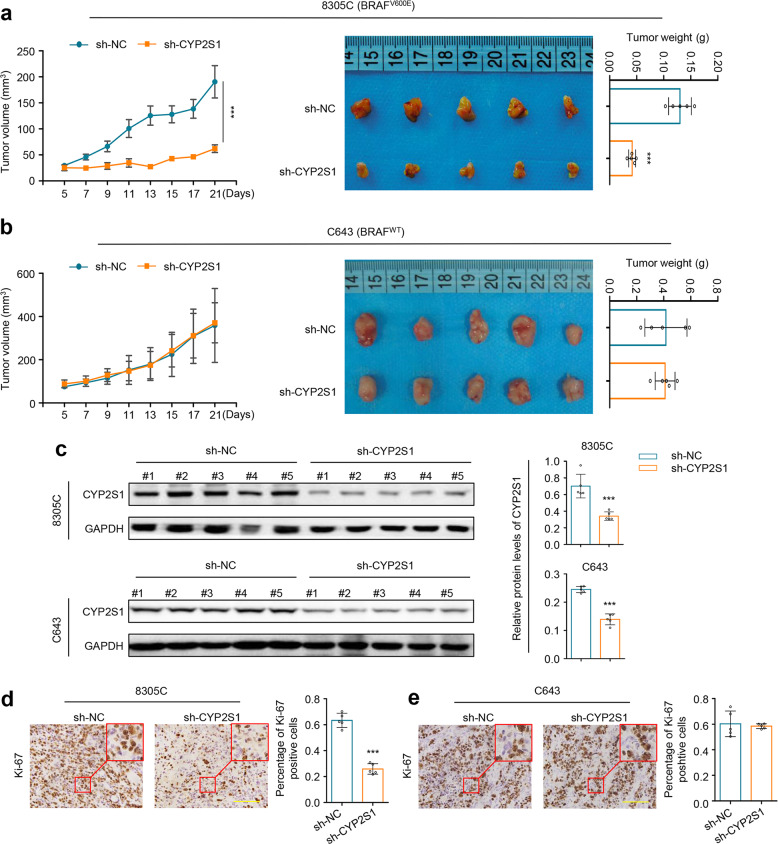
Validation of CYP2S1 as a synthetic lethal partner of BRAF^V600E^ in vivo. **a**, **b** Establishment of xenograft tumor models by subcutaneous inoculation of 8305C and C643 cells stably knocking down CYP2S1 or control cells (*n* = 5 per group). Left panels show time courses of tumor growth. Representative images of xenograft tumors and statistical analysis of tumor weight were shown in right panels. **c** Validation of CYP2S1 knockdown in xenograft tumor tissues by western blot analysis (left panels). GAPDH was used as a loading control. The density of CYP2S1 proteins on western blot was normalized to GAPDH, and statistical analysis was shown in right panel. **d**, **e** The levels of Ki-67 proteins in the indicated xenograft tumors by IHC assay (left panels). Statistical analysis of the percentage of Ki-67 positive cells was shown in right panels. Scale bars, 200 µm. Error bars represent SD. ***P < 0.001

To validate the above conclusions in vivo, we established tumor xenografts in nude mice by injecting 8305C cells harboring BRAF^V600E^ or C643 cells harboring wild-type BRAF. The results indicated that, compared to the controls, CYP2S1 depletion led to a significant reduce in the growth rate and weight of 8305C cell-derived xenograft tumors (Fig. 3a), while almost did not affect the growth of C643 cell-derived xenograft tumors (Fig. 3b). At the end of experiments, we performed western blot and IHC assays to confirm the inhibition of CYP2S1 expression in xenograft tumors (Fig. 3c, Supplementary Fig. 4). Next, we determined the effect of CYP2S1 depletion on cell proliferation in xenograft tumors by staining Ki-67. The results showed that, relative to the controls, CYP2S1 depletion remarkably decreased the percentage of Ki-67 positive cells in 8305C cell-derived xenograft tumors (Fig. 3d), while almost did not change Ki-67 levels in C643 cell-derived xenograft tumors (Fig. 3e), further supporting the above in vitro findings.

### BRAF^V600E^-mediated MAPK/ERK cascade increases CYP2S1 expression in thyroid cancer cells

**Fig. 4:**
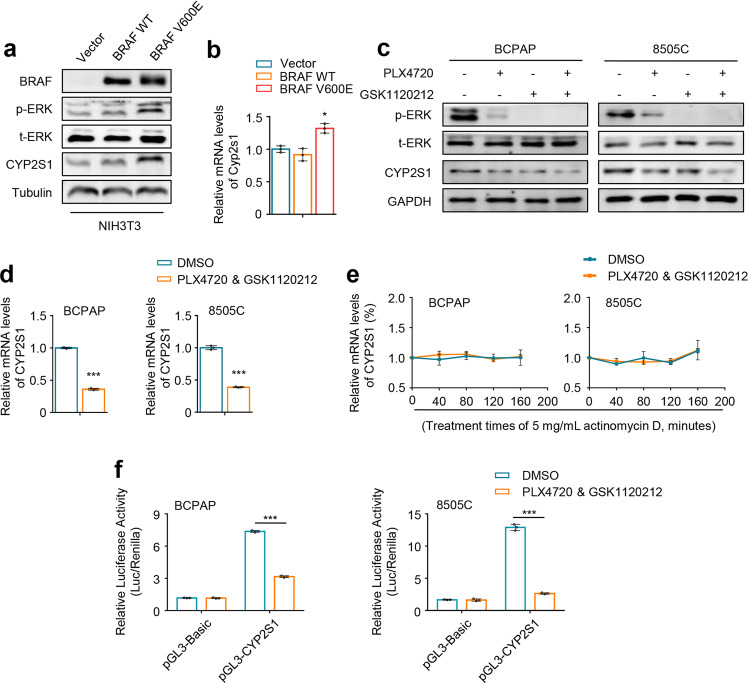
Upregulation of CYP2S1 expression by BRAF^V600E^-mediated MAPK/ERK cascade. **a** Western blot analysis was performed to validate ectopic expression of human wild-type (WT) and V600E mutant BRAF in NIH3T3 cells, and investigate their effect on ERK phosphorylation (p-ERK) and CYP2S1 expression. Tubulin was used as a loading control. **b** qRT-PCR was performed to evaluate the effect of ectopic expression of wild-type BRAF and BRAF^V600E^ on mRNA levels of Cyp2s1 in NIH3T3 cells. 18S rRNA was used as a reference gene. BCPAP and 8505C cells were treated with 1 μM PLX4720 alone or in combination with 500 nM GSK1120212 for 24 h. **c** Western blot analysis was performed to test their effect on ERK phosphorylation (p-ERK) and CYP2S1 expression. GAPDH was used as a loading control. **d** BCPAP and 8505C cells were treated with a combination of 1 μM PLX4720 and 500 nM GSK1120212 for 24 h, and qRT-PCR was then performed to evaluate its effect on mRNA levels of CYP2S1. 18S rRNA was used as a reference gene. **e** BCPAP and 8505C cells were treated with a combination of 1 μM PLX4720 and 500 nM GSK1120212 for 24 h, followed by 5 mg/mL actinomycin D for indicated time, and qRT-PCR was then performed to evaluate its effect on mRNA levels of CYP2S1. 18S rRNA was used as a reference gene. **f** Dual-luciferase reporter system was used to evaluate the effect of a combined treatment of PLX4720 and GSK1120212 on the activity of CYP2S1 promoter in BCPAP and 8505C cells. Renilla luciferase was used as an internal control. Error bars represent SD. *P < 0.05; ***P < 0.001

The above results showed that CYP2S1 was significantly upregulated in BRAF^V600E^-mutated PTCs relative to BRAF wild-type ones. This observation suggests that BRAF^V600E^-mediated MAPK/ERK signaling may be involved in regulating CYP2S1 expression in thyroid cancers. First, we ectopically expressed human wild-type BRAF and BRAF^V600E^ in NIH3T3 cells. The results showed that, compared to empty vector, wild-type BRAF almost did not affect the activity of MAPK/ERK signaling and CYP2S1 expression, while BRAF^V600E^ strongly enhanced ERK phosphorylation and increased CYP2S1 expression at both protein and mRNA levels (Fig. 4a, b).

On the other hand, we treated BRAF^V600E^-mutated thyroid cancer lines BCPAP and 8505C with 1 μM PLX4720 (a selective inhibitor of BRAF^V600E^) alone or in combination with 500 nM GSK1120212 (MEK1/2 inhibitor) for 24 h. The results showed that both PLX4720 and GSK1120212 effectively suppressed ERK phosphorylation and decreased protein expression of CYP2S1, particularly a combined treatment of these two drugs (Fig. 4c), also supported by qRT-PCR results that mRNA expression of CYP2S1 was significantly inhibited by PLX4720 in combination with GSK1120212 (Fig. 4d).

To further determine whether activated ERK promotes CYP2S1 expression by stabilizing mRNA or enhancing its transcription, we used actinomycin D to monitor the mRNA decay when BCPAP and 8505C cells were treated with PLX4720 and GSK1120212 or DMSO. As shown in Fig. 4e and Supplementary Fig. 5, the treatment of BCPAP and 8505C cells with PLX4720 and GSK1120212 almost did not affect the mRNA stabilization of CYP2S1. In addition, a combined treatment of PLX4720 and GSK1120212 significantly inhibited the activity of CYP2S1 promoter compared to the control in BCPAP and 8505C cells (Fig. 4f). These data indicate that BRAF^V600E^ enhances CYP2S1 transcription at least partially through activating MAPK/ERK signaling pathway.

### BRAF^V600E^ promotes CYP2S1 expression via an AHR-dependent pathway

**Fig. 5:**
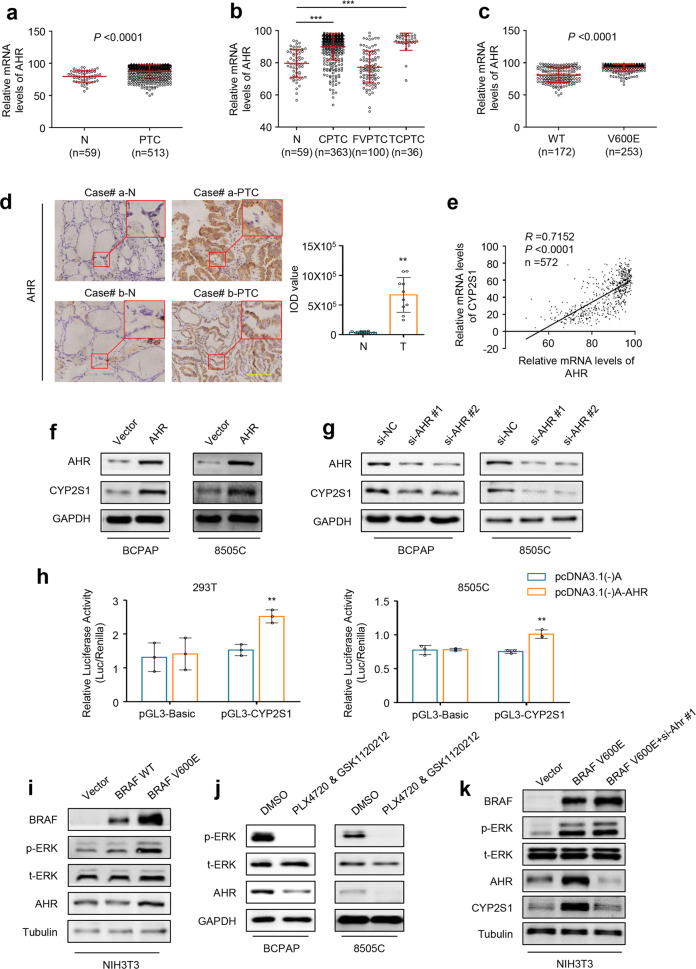
AHR-dependent upregulation of CYP2S1 by BRAF^V600E^-mediated MAPK/ERK signaling. **a** High expression of AHR in PTCs compared to normal thyroid tissues (N) from TCGA database. **b** CYP2S1 expression in different histological subtypes of PTCs. CPTC, conventional PTC; FVPTC, follicular variants PTC; TCPTC, tall-cell PTC. **c** High expression of AHR in BRAF^V600E^ mutated PTCs (V600E) compared to BRAF wild-type ones (WT). **d** Evaluation of AHR expression in 10 PTCs and their matched noncancerous tissues (N) by IHC staining. Representative IHC images were shown in left panel. Scale bars, 200 µm. Quantitative illustration of AHR proteins was shown in right panel. **e** The relationship between mRNA expression of CYP2S1 and AHR in PTCs from TCGA database. Western blot analysis was performed to evaluate the effects of ectopic expression of AHR (**f**) and AHR knockdown (**g**) on CYP2S1 expression in BCPAP and 8505C cells. GAPDH was used as a loading control. **h** Dual-luciferase reporter system was used to evaluate the effect of ectopic expression of AHR on promoter activity of CYP2S1 in 293T and 8505C cells. Renilla luciferase was used as an internal control. **i** Western blot analysis was performed to evaluate the effect of ectopic expression of wild-type BRAF and BRAF^V600E^ on ERK phosphorylation (p-ERK) and AHR expression in NIH3T3 cells. Tubulin was used as a loading control. **j** BCPAP and 8505C cells were treated with a combination of 1 μM PLX4720 and 500 nM GSK1120212 for 24 h, and western blot analysis was then performed to evaluate its effect on ERK phosphorylation (p-ERK) and AHR expression. GAPDH was used as loading control. **k** AHR was knocked down in NIH3T3 cells expressing BRAF^V600E^, and western blot analysis was then performed to determine whether BRAF^V600E^ regulated CYP2S1 expression via AHR. Tubulin was used as a loading control. Error bars represent SD. **P < 0.01; ***P < 0.001

It should be noted that although CYP2S1 is classified into the CYP2 family based on its amino acid sequence identity,^19^ it exhibits typical features of CYP1 family members such as induced by dioxin via AHR.^10,20^ Thus, we suppose that BRAF^V600E^ regulates CYP2S1 expression in thyroid cancer cells probably by AHR. First, we analyzed AHR expression using TCGA database, and found that its expression pattern was similar to that of CYP2S1 (Fig. 5a–c). AHR expression was significantly upregulated in CPTCs and TCPTCs compared to control subjects (Fig. 5b). Moreover, BRAF^V600E^-mutated PTCs exhibited higher expression of AHR than BRAF wild-type ones (Fig. 5c). The IHC results further confirmed high expression of AHR in PTCs compared to control subjects (Fig. 5d).

Notably, we found a close relationship between mRNA expression of AHR and CYP2S1 in PTCs from TCGA dataset (Fig. 5e), suggesting that AHR may be involved in regulating CYP2S1 transcription. To prove this, we ectopically expressed AHR in BCPAP and 8505C cells, and found that ectopic expression of AHR strongly increased CYP2S1 expression (Fig. 5f). Conversely, AHR knockdown in these cells decreased CYP2S1 expression (Fig. 5g). To determine whether AHR directly regulates the activity of CYP2S1 promoter, we constructed luciferase reporter plasmid containing CYP2S1 promoter. As expected, ectopic expression of AHR significantly enhanced the activity of CYP2S1 promoter in 293T and 8505C cells (Fig. 5h). These data indicate that CYP2S1 is a potential downstream target of AHR.

Considering a positive association of BRAF^V600E^ mutation with AHR expression, we thus speculate that BRAF^V600E^-mediated MAPK/ERK signaling can upregulate AHR expression, thereby enhancing CYP2S1 transcription. First, we ectopically expressed human wild-type BRAF and BRAF^V600E^ in NIH3T3 cells, and found that BRAF^V600E^ clearly increased ERK phosphorylation and AHR expression compared to empty vector and wild-type BRAF (Fig. 5i). Meanwhile, we also observed that BRAF^V600E^ significantly upregulated a well-known downstream target of AHR, Cyp1b1, relative to empty vector, and wild-type BRAF (Supplementary Fig. 6a). Conversely, we treated BCPAP and 8505C cells with 1 μM PLX4720 in combination with 500 nM GSK1120212 for 24 h. This caused a strong inhibition of ERK phosphorylation and a decreased expression of AHR and its target CYP1B1 (Fig. 5j, Supplementary Fig. 6b).

To figure out whether BRAF^V600E^ induces CYP2S1 expression via AHR, we knocked down AHR expression in NIH3T3 cells ectopically expressing BRAF^V600E^. The results expectedly showed that BRAF^V600E^ dramatically increased the expression of AHR and CYP2S1 compared to empty vector; however, BRAF^V600E^-mediated Cyp2s1 upregulation could be reversed by AHR depletion (Fig. 5k), indicating that BRAF^V600E^ increases CYP2S1 expression via an AHR dependent pathway.

### A positive feedback loop between CYP2S1 and AHR

**Fig. 6:**
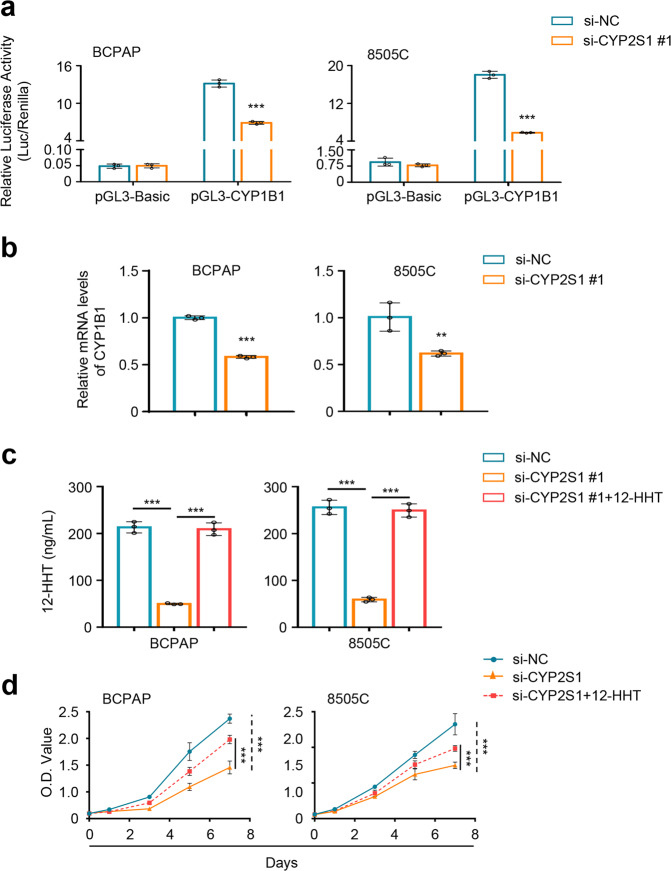
AHR/CYP2S1 feedback loop causing synthetic lethal interaction between CYP2S1 and BRAF^V600E^. **a** Dual-luciferase reporter system was used to evaluate the effect of CYP2S1 knockdown on promoter activity of a downstream target of AHR, CYP1B1, in BCPAP and 8505C cells. Renilla luciferase was used as an internal control. **b** qRT-PCR was performed to evaluate the effect of CYP2S1 knockdown on mRNA levels of CYP1B1. 18S rRNA was used as a reference gene. **c** HPLC was used to analyze 12-HHT levels in the supernatant of BCPAP and 8505C cells. **d** The effect of CYP2S1 knockdown and 12-HHT restoration on the proliferation of BCPAP and 8505C cells was monitored by MTT assay. Error bars represent SD. Each experiment was performed in triplicate. **P < 0.01; ***P < 0.001

There is evidence for the existence of a positive regulatory loop between CYP1 and AHR by the metabolic substrates,^21^ we thus suppose that it exists similar regulatory loop between CYP2S1 and AHR. To validate this, we constructed luciferase reporter plasmid containing CYP1B1 promoter, which is a downstream target of AHR, and performed dual-luciferase reporter assays. The results showed that CYP2S1 knockdown in BCPAP and 8505C cells significantly reduced promoter activity of CYP1B1 compared to the control (Fig. 6a). Accordingly, we also observed that downstream target of AHR, CYP1B1, was significantly downregulated upon CYP2S1 depletion (Fig. 6b). These results support the existence of the CYP2S1-AHR feedback loop in the BRAF^V600E^-mutated thyroid cancer cells.

**Fig. 7:**
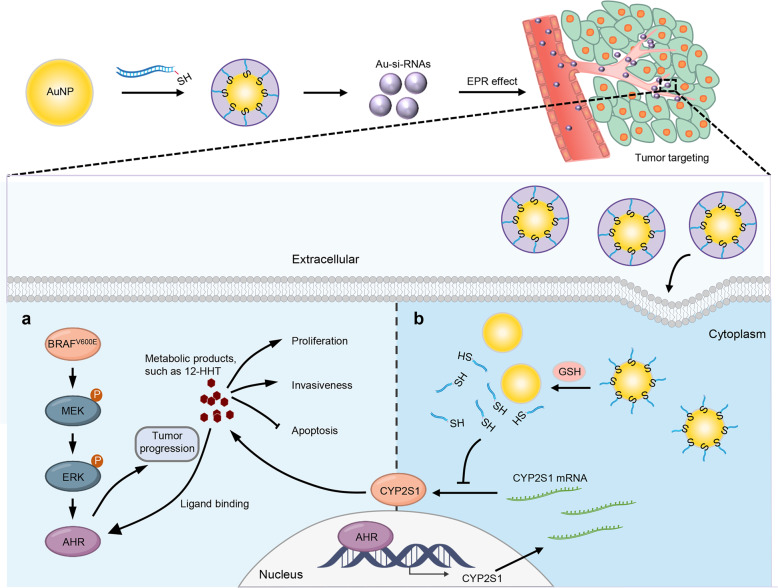
A schematic model for synthetic lethal interaction between CYP2S1 and BRAF^V600E^ and corresponding therapeutic strategy. Briefly, in the BRAF^V600E^-mutated thyroid cancer cells, activated MAPK/ERK signaling increases AHR expression, and subsequently promotes AHR-mediated CYP2S1 transcription. CYP2S1 catalyzes its endogenous substrates to carcinogenic metabolites such as 12-HHT, contributing to malignant phenotypes of cancer cells. On the other hand, some of metabolites may act as endogenous ligands of AHR to enhance its transcriptional activity, thereby forming a positive AHR/CYP2S1 feedback loop. This feedback loop significantly enhances oncogenic role of BRAF^V600E^ in thyroid cancer cells, causing the formation of synthetic lethal interaction between CYP2S1 and BRAF^V600E^ (**a**). A collaurum-based siRNA delivery system was designed to target CYP2S1 (**b**)

Next, we choose one of the classical CYP2S1 metabolic substrates, 12-HHT,^12^ and tested its biological role in BRAF^V600E^-mutated thyroid cancer cells. Using high performance liquid chromatography (HPLC), we demonstrated that CYP2S1 knockdown significantly reduced 12-HHT levels in the supernatants of BCPAP and 8505C cells (Supplementary Fig. 7a, Fig. 6c), while CYP2S1 knockdown in C643 cells almost did not affect 12-HHT levels (Supplementary Fig. 7b). As expected, the restoration of 12-HHT could partially reverse inhibitory effect of CYP2S1 knockdown in cell viability by adding 12-HHT to the supernatants of BCPAP and 8505C cells knocking down CYP2S1 (Fig. 6c, d). However, the restoration of 12-HHT did not affect transcriptional activity of AHR (data not shown), indicating that 12-HHT is not an endogenous AHR ligand. Considering that metabolic substrates of CYP2S1 are not well identified, endogenous ligands of AHR need to be further investigated in the future. Collectively, our data indicate the existence of the CYP2S1-AHR feedback loop, contributing to malignant phenotypes of BRAF^V600E^-driven thyroid cancers, and also make rationalization of the synthetic lethal interaction between CYP2S1 and BRAF^V600E^ (Fig. 7a).

### CYP2S1 is a potential therapeutic target in BRAF^V600E^-driven thyroid cancers

**Fig. 8:**
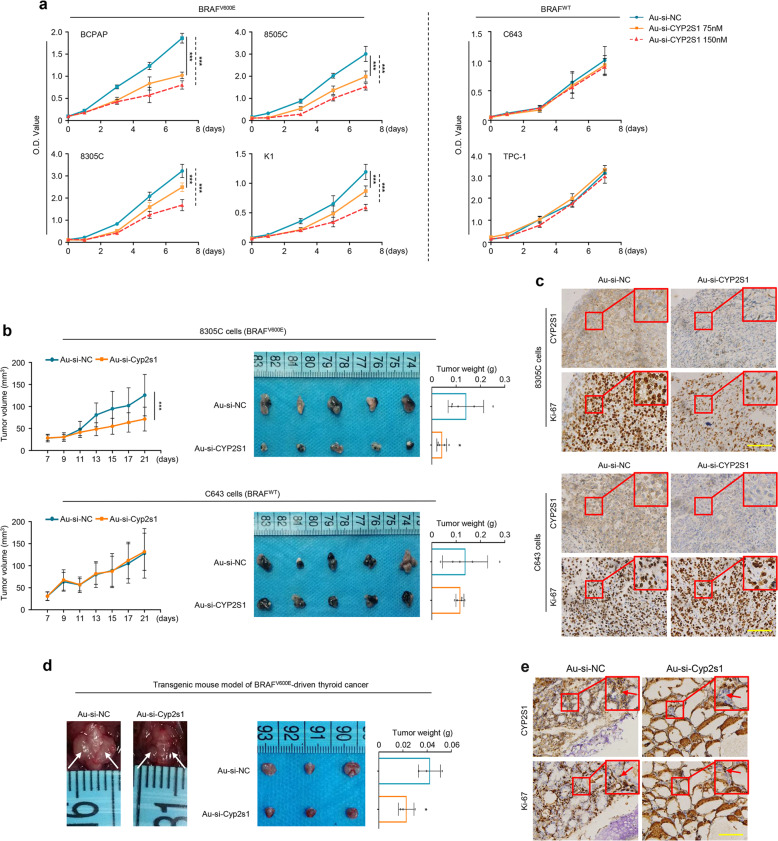
Targeting CYP2S1 is an effective therapeutic strategy for BRAF^V600E^ mutated thyroid cancer cells. **a** Time courses of cell proliferation upon CYP2S1 knockdown in the indicated cell lines were determined by MTT assay. **b** Xenograft tumor models were established by subcutaneous inoculation of 8305C (5 × 10^6^) or C643 (3 × 10^6^) cells, and mice were then randomized to two groups (Au-si-CYP2S1 and Au-si-NC; *n* = 5/group), respectively. Left panels show time courses of tumor growth in mice with the indicated treatments. Representative images of xenograft tumors and statistical analysis of tumor weight were shown in right panels. **c** Evaluation of CYP2S1 and Ki-67 expression in the indicated xenograft tumors by IHC assay. Scale bars, 200 µm. **d** Transgenic mouse models of thyroid cancer were established, and similarly treated with as mentioned above. Representative images of thyroid tumors in the indicated groups were shown in left panel. Right panel represents mean tumor weight (*n* = 3/group). **e** Evaluation of CYP2S1 and Ki-67 expression in the indicated tumor tissues by IHC assay. Scale bars, 200 µm. Error bars represent SD. *P < 0.05; **P < 0.01; ***P < 0.001

The above findings indicate that CYP2S1 may be a potential therapeutic target in BRAF^V600E^-driven thyroid cancers. To prove this, we first established a collaurum-based siRNA delivery system, which has been demonstrated to be an effective strategy for cancer therapy in vitro and in vivo.^22–24^ Based on our design, Au-si-RNAs can be effectively accumulated in tumor sites by enhanced permeability and retention (EPR) effect, and enter cancer cells via endocytosis. Under intracellular glutathione (GSH), si-CYP2S1 can be released to target and downregulate CYP2S1 (Fig. 7b). As shown in Supplementary Fig. 8a and b, successful synthesis of Au-si-RNAs were verified by dynamic light scattering and ultraviolet–visible spectra absorption. Their size is appropriate to extravasate the leaky pore of tumor vasculature via EPR effect.^25–27^ Expectedly, Au-si-CYP2S1 could effectively suppress CYP2S1 expression in both BRAF^V600E^-mutated and BRAF wild-type thyroid cancer cells compared to the control (Supplementary Fig. 8c). Similarly, Au-si-CYP2S1 selectively inhibited cell proliferation in BRAF^V600E^-mutated thyroid cancer cells, but not in BRAF wild-type ones (Fig. 8a). Besides, Au-si-CYP2S1 also enhanced the antitumor efficacy of the doxorubicin (DOX), a traditional chemotherapeutic drug, compared to the control (Supplementary Fig. 9).

Next, to further validate therapeutic potential of Au-si-CYP2S1 in vivo, we established xenograft and transgenic mouse models of thyroid cancer. As expected, compared to Au-si-NC, Au-si-CYP2S1 remarkably inhibited the growth and weight of 8305C cell-derived xenograft tumors, but not C643 cell-derived xenograft tumors (Fig. 8b). This was further supported by Ki-67 and CYP2S1 staining (Fig. 8c, Supplementary Fig. 10). Besides, we observed that Au-si-NC treatment significantly decreased body weight of mice compared to Au-si-CYP2S1 treatment in 8305C cell-derived xenograft models, but not in C643 cell-derived xenograft models (Supplementary Fig. 11).

We also designed Au-si-Cyp2s1 to target Cyp2s1 in NIH3T3 cells and transgenic model of thyroid cancer. The results showed that Au-si-Cyp2s1 effectively suppressed CYP2S1 expression in NIH3T3 cells compared to the control (Supplementary Fig. 12). In addition, Au-si-Cyp2s1 obviously inhibited tumor growth and weight in transgenic model of thyroid cancer (Fig. 8d), also supported by Ki-67 and CYP2S1 staining (Fig. 8e, Supplementary Fig. 13). Importantly, Au-si-RNAs almost did not cause obvious side effects on the liver or kidney of mice in both xenograft and transgenic mouse models (Supplementary Fig. 14). Taken together, our data indicate that CYP2S1 is an effective therapeutic target in BRAF^V600E^-driven thyroid cancers.

## Discussion

BRAF^V600E^ mutation occurs exclusively in PTCs and some ATCs arising from PTCs,^28^ and it is now recognized as the most common genetic alteration in thyroid cancers.^29^ The tumorigenic role of BRAF^V600E^ mutation had been proved through thyroid-specific knock-in of BRAF^V600E^, resulting in aggressive PTC.^30^ Thyroid cancers carrying BRAF^V600E^ mutation always indicate poor clinical outcomes and are more likely to resistant to traditional therapies.^31,32^ Although the MAPK/ERK signaling rebound following the applying of BRAF^V600E^ specific inhibitors limits their clinical use, targeting BRAF^V600E^ is still promising.

In this study, we found that CYP2S1 was highly expressed in PTCs relative to control subjects, particularly in CPTCs and TCPTCs, and its expression was strongly associated with BRAF^V600E^ mutation. By a series of in vitro and in vivo functional studies, we demonstrated that CYP2S1 knockdown selectively inhibited cell proliferation, colony formation, migration, invasion, and tumorigenic potential in nude mice, and induced cell apoptosis in BRAF^V600E^-mutated thyroid cancer cells, but not in BRAF wild-type ones. These results indicate that CYP2S1 may serve as a potential synthetic lethal partner of BRAF^V600E^ in thyroid cancers.

As a member of the CYP family, it is possible that oncogenic role of CYP2S1 is dependent on its metabolic capacity. Current knowledge of CYP2S1 in endogenous function is largely related to eicosanoids metabolism, and some of its metabolic products have been identified, including 12-HHT, 5-oxo-eicosatetraenoic acid (5-oxoETE), 12-oxoETE, 15-oxoETE, etc.^12^ Some of them have been demonstrated to be involved in tumor progression, such as 12-HHT and 5-oxoETE,^33,34^ as supported by our data that CYP2S1 knockdown in BCPAP and 8505C cells dramatically reduced 12-HHT levels, and the restoration of 12-HHT could partially reverse inhibitory effect of CYP2S1 knockdown in cell viability. Besides, our data demonstrated that BRAF^V600E^ promotes CYP2S1 transcription by activating MAPK/ERK cascade. Thus, we suppose that increased expression of CYP2S1 in BRAF^V600E^-mutated thyroid cancer cells lead to the concentration of certain metabolic products such as 12-HHT and 5-oxoETE, thereby promoting malignant phenotypes of cancer cells.

Next, we attempted to reveal the mechanism underlying the formation of synthetic lethal interaction between CYP2S1 and BRAF^V600E^. First, we demonstrated that BRAF^V600E^ increased CYP2S1 expression via an AHR-dependent pathway. As a ligand-activated transcription factor, AHR was discovered as a receptor of 2,3,7,8-tetrachlorodibenzo-p-dioxin (dioxin),^35^ and was reported to be highly expressed in different types of cancer, contributing to malignant phenotypes of cancer cells.^36–38^ Furthermore, there is study showing that AHR can transcriptionally regulate CYP enzymes.^11,20,39^ As supported, our study demonstrated that AHR indeed regulated CYP2S1 transcription, and further identified CYP2S1 as a potential downstream target of AHR. In addition, although our data showed that BRAF^V600E^ increased AHR expression by the MAPK/ERK signaling, the exact mechanism remains elusive. For example, it will need to be determined whether BRAF^V600E^ is involved in regulating AHR transcription or affects its protein stability.

It should be noted that, in our study, CYP2S1 knockdown can decrease transcriptional activity of AHR and the expression of its well-known downstream target CYP1B1, indicating that CYP2S1 in turn enhances transcriptional activity of AHR. This will form a positive CYP2S1-AHR regulatory loop. This was supported by a previous study that 12(R)-hydroxy-5(Z),8(Z),10(E),14(Z)-eicosatetraenoic acid [12(R)-HETE], as a metabolite of CYP2S1, could be converted to an endogenous AHR ligand to enhance its transcriptional activity.^40^ This positive feedback loop will further amplify the BRAF^V600E^/AHR/CYP2S1 oncogenic signal, strongly promoting malignant progression of BRAF^V600E^-mutated thyroid cancer cells. These findings can partially illustrate the mechanism underlying synthetic lethal interaction between CYP2S1 and BRAF^V600E^ in thyroid cancers. More importantly, we established a collaurum-based siRNA delivery system, and successfully delivered CYP2S1-specific siRNA to tumor site in both xenograft and transgenic mouse models using this system. As expected, our data demonstrated that Au-si-CYP2S1 selectively inhibited BRAF^V600E^-mutated thyroid cancer cells, while almost did not affect the growth of BRAF^V600E^ wild-type ones. This raised the possibility that targeting CYP2S1 may be an effective strategy for BRAF^V600E^-mutated thyroid cancers.

In summary, we find that BRAF^V600E^-mediated MAPK/ERK cascade increases CYP2S1 expression via an AHR dependent pathway, while certain metabolites of CYP2S1 may in turn act as endogenous AHR ligands to activate its transcriptional activity. This AHR/CYP2S1 feedback loop strongly amplifies oncogenic role of BRAF^V600E^ in thyroid cancer cells, thereby causing synthetic lethal interaction between CYP2S1 and BRAF^V600E^. Thus, our data demonstrate that CYP2S1 is a potential synthetic lethal partner of BRAF^V600E^, and also provide an alternative therapeutic strategy for BRAF^V600E^-mutated thyroid cancers by targeting CYP2S1.

## Materials and methods

### Clinical samples

With the approval of the Institutional Review Board and Human Ethics Committee of the First Affiliated Hospital of Xi’an Jiaotong University School, ten paraffin-embedded primary PTCs and their matched noncancerous thyroid tissues (control subjects) were obtained from this hospital. Informed consent was provided to all patients prior to conducting this study. None of the patients received any therapeutic intervention before the surgery. All of the excised tissues were histologically examined by two senior pathologists at the Department of Pathology of the Hospital based on World Health Organization (WHO) criteria.

### Cell culture and drug treatments

Human thyroid cancer cell lines, such as BCPAP, 8305C, 8505C, K1, and TPC-1 were kindly provided by Dr. Haixia Guan (The First Affiliated Hospital of China Medical University, Shenyang, China). Human thyroid cancer cell line C643 was provided by Dr. Lei Ye (Ruijin Hospital, Shanghai, China). Human embryonic kidney cell line 293T and mouse fibroblast cell line NIH3T3 were obtained from ATCC (Rockville, MD). All cell lines were routinely cultured at 37 °C in RPMI 1640 or DMEM medium with 10% fetal bovine serum. In some experiments, cells were treated with 1 μM BRAF kinase inhibitor PLX4720 (Selleck Chemicals, TX, USA), 500 nM MEK1/2 inhibitor GSK1120212 (Selleck Chemicals, TX, USA), 5 mg/mL actinomycin D (Selleck Chemicals, TX, USA), 30 nM DOX (Selleck Chemicals, TX, USA) for the indicated times. All inhibitors were dissolved in dimethylsulfoxide (DMSO), aliquoted and stored at −80 °C until use. The same volume of DMSO was used as control.

### RNA interference, lentivirus transfection, and expression plasmids

Control siRNA and specific siRNAs targeting CYP2S1 (human), Cyp2s1 (mouse), AHR (human), and Ahr (mouse) were obtained from Gene Pharma (Shanghai, China), the sequences were presented in supplementary Table 1. Cells were then transfected with 50 nM siRNAs at ~50% confluent using Lipofectamine 2000 (Invitrogen, NY, USA).

Lentivirus expressing shRNA targeting CYP2S1 and control lentivirus were obtained from HanBio Biotechnology Co., Ltd (Shanghai, China), and their sequences were also presented in Supplementary Table 1. When cells became ~50% confluent, they were infected with the above lentiviruses at multiplicity of infection of 20–60, and then selected with 2 μg/ml puromycin for two weeks.

The plasmids expressing wild-type BRAF and BRAF^V600E^ mutant, and the empty vector (pEFm6) were kindly provided by Dr. Richard Marials (Cancer Research UK Manchester Institute, Manchester, UK). The plasmid expressing AHR was created by cloning open reading frame of AHR gene into mammalian expression vector pcDNA3.1(−)A, and the sequences were presented in Supplementary Table 2. Cells were transfected with the plasmids at ~70% confluent using Lipofectamine 2000 (Invitrogen, NY, USA).

### Cell proliferation, colony formation, migration, invasion, and apoptosis assays

The detailed procedures were similarly performed as described previously.^41,42^

### Western blot analysis

The protocol was described as previously.^42^ The antibody information was presented in Supplementary Table 3.

### RNA extraction and quantitative RT-PCR (qRT-PCR)

RNA extraction, cDNA synthesis and qRT-PCR were performed as previously described.^43^ 18S rRNA was used as a reference gene to normalize mRNA expression of the indicated genes. The primer sequences were presented in Supplementary Table 4. Each sample was repeated in triplicate.

### Dual-luciferase reporter assay

pGL3-CYP2S1-Luc and pGL3-CYP1B1-Luc were created by cloning promoter regions of CYP2S1 and CYP1B1 genes into pGL3-Basic luciferase vector (Promega Corp., WI, USA), and the constructs were then verified by Sanger sequencing. To determine regulatory role of CYP2S1 promoter by AHR, 293T, or 8505C cells expressing AHR or control cells were co-transfected with luciferase reporter plasmids (pGL3-Basic or pGL3-CYP2S1-Luc) and pRL-TK plasmids (Promega Corp., WI, USA). To further determine the effect of CYP2S1 depletion on AHR-mediated transcriptional activation of CYP1B1, BCPAP, and 8505C cells were firstly transfected with siRNAs targeting CYP2S1 or control siRNA, and then co-transfected with luciferase reporter plasmids (pGL3-Basic or pGL3-CYP1B1-Luc) and pRL-TK plasmids. Cell were collected 48 h post-transfection, and luciferase activities were measured using the dual-luciferase reporter assay system (Promega Corp., WI, USA). Primer sequences for dual-luciferase reporter plasmids were presented in Supplementary Table 5.

### High performance liquid chromatography

The supernatant of 2 × 10^7^ cells was collected, and then the HPLC were performed to analyze 12-HHT levels as described previously.^44^

### Collaurum-based siRNA delivery

Thiolated siRNAs targeting CYP2S1/Cyp2s1 and control siRNA were obtained from RiboBio Co., Ltd. (Guangzhou, China). siRNA conjunct collaurum delivery system (Au-si-CYP2S1 or Au-si-NC) were synthesized and verified as described previously.^21,22,45^ Cells were treated with si-CYP2S1 or Au-si-NC at the indicated concentrations for 48 h, and cells were then harvested and subjected to further experiments.

### Animal studies

Four to five-week-old female athymic nude mice purchased from SLAC laboratory Animal Co., Ltd. (Shanghai, China) were housed in a specific pathogen-free environment, and then randomly divided into four groups (*n* = 5/group). Tumor xenografts were established by subcutaneous inoculation of 8305C (5 × 10^6^) and C643 (3 × 10^6^) cells stably knocking down CYP2S1 or control cells into flanks of nude mice Tumor size were measured by a Vernier caliper every other day since 5 days after injection, and tumor volumes were calculated by the formula: Tumor volume = length × width^2^ × 0.5. The mice were sacrificed at 21 days after injection and tumors were then weighted and harvested for further exams.

The mice with xenografted tumors drived from 8305C or C643 were randomized to two groups (Au-si-CYP2S1 and Au-si-NC; *n* = 5/group) when tumors reached ~30 mm^3^. Body weights were measured over the course of the study on a standard laboratory scale. The mice were treated every other day with Au-si-CYP2S1 or Au-si-NC (100 μL, 2 × 10^−6^ M Au-siRNAs; ~0.7 mg siRNAs kg^−1^) for a total of seven treatments via tail vein.

Transgenic mouse strains *TPO-CreER* and *Braf*^*CA*^ were kindly provided by Profs. Tyler Jacks (Massachusetts Institute of Technology, USA) and Martin McMahon (University of California, USA). *Tp53*^*F/F*^ mouse strain was purchased from The Jackson Laboratory (stock no. 008462). Thyroid cancer mouse model was similarly established as previously described.^46^ Au-siRNAs were administered 8 weeks following tumor induction with the same dose schedule as xenograft model. The mice were sacrificed after a 5-week treatment, and tumors were then weighted and harvested for further exam.

### Immunohistochemistry (IHC)

IHC assay was performed to evaluate the expression of CYP2S1, AHR and Ki-67 in xenograft tumor sections as described previously.^42^ Protein expression was quantitated by integral optical density using Image-pro plus 6.0 (Media Cybernetics, USA). Each stained section was evaluated under the same magnification, light brightness and exposure intensity. The evaluation of the percentage of Ki-67 positive cells was conducted by calculating the number of the positive cells in ten microscopic fields from each group.

### Statistical analysis

All statistical analyses were conducted using the SPSS statistical package (16.0, SPSS Inc. Chicago, IL). Unpaired student’s *t* test was used to compare the means of two groups of data. One-way analysis of variance (ANOVA) followed by Bonferroni’s multiple comparison test was used to compare differences between groups. All values were expressed as the mean ± standard deviation. All values with *P* < 0.05 were considered significantly.

## Supplementary information

Supplementary Material

## Data Availability

The authors confirm that the data supporting the findings of this study are available within the article and its supplementary materials.
