# Supplementary material for: CYP2S1 is a synthetic lethal target in BRAFV600E-driven thyroid cancers
Source: Signal Transduct Target Ther. 2020 Sep 11;5:191. doi: 10.1038/s41392-020-00231-6 (PMC7483764; doi:10.1038/s41392-020-00231-6)
Supplement: Supplementary file 1 — Supplementary Material [file 41392_2020_231_MOESM1_ESM.docx]

Supplementary Materials for

**CYP2S1 is a synthetic lethal target in BRAF^V600E^-driven thyroid cancers**

Yiqi Li^1,#^, Xi Su^1,#^, Chao Feng^1^, Siyu Liu^1^, Haixia Guan^2^, Yue Sun^3^, Nongyue He^4,*^, Meiju Ji^5,*^, and Peng Hou^1,6,*^

^1^Department of Endocrinology, The First Affiliated Hospital of Xi’an Jiaotong University, Xi’an 710061, P.R. China

^2^Department of Endocrinology and Metabolism, The First Affiliated Hospital of China Medical University, Shenyang 110001, P.R. China

^3^Philips Institute for Oral Health Research, Virginia Commonwealth University, Richmond, VA 23298, USA

^4^State Key Laboratory of Bioelectronics, Southeast University, Nanjing 210096, P.R. China

^5^Center for Translational Medicine, The First Affiliated Hospital of Xi’an Jiaotong University, Xi’an 710061, P.R. China

^6^Key Laboratory for Tumor Precision Medicine of Shaanxi Province, The First Affiliated Hospital of Xi’an Jiaotong University, Xi’an 710061, P.R. China.

**^*^Correspondence to:**

Peng Hou: phou@xjtu.edu.cn

Meiju Ji: mjji0409@163.com

Nongyue He: nyhe1958@163.com

**Supplementary Materials for this manuscript include the following:**

Fig. S1. Increased expression of HMGCL, HMGCS1, CYP39A1, CYP2C9, CYP2E1, CYP2J2 and CSGLCA-T in PTCs.

Fig. S2. Expression of 8 metabolism related genes in BRAF^V600E^ PTCs.

Fig. S3. Expression of CYP2S1 in different type of BRAF^V600E^ cancers.

Fig. S4. Validation of CYP2S1 expression in the xenograft tumors.

Fig. S5. Western blot to verify the inhibition of ERK phosphorylation.

Fig. S6. Upregulation of CYP1B1 expression by BRAF^V600E^-mediated MAPK/ERK cascade.

Fig. S7. Quantitative analysis of 12-HHT levels in thyroid cancer cells.

Fig. S8. Validation of the characters and function of Au-si-RNAs.

Fig. S9. Au-si-CYP2S1 enhanced the antitumor activities of DOX.

Fig. S10. Statistical analysis of CYP2S1 expression and the percentage of Ki-67 positive cells.

Fig. S11. The body weight of mice.

Fig. S12. Validation of the inhibition of Cyp2s1 expression in NIH3T3 cell.

Fig. S13. Statistical analysis of Cyp2s1 expression and the percentage of Ki-67 positive cells.

Fig. S14. H&E-stained of liver and kidney.

Table S1. siRNAs and shRNAs used in this study.

Table S2. The primers used in this study for plasmid construction.

Table S3. Antibodies used in this study.

Table S4. qRT-PCR primers used in this study.

Table S5. The primers used in this study for luciferase reporter plasmid construction.

**
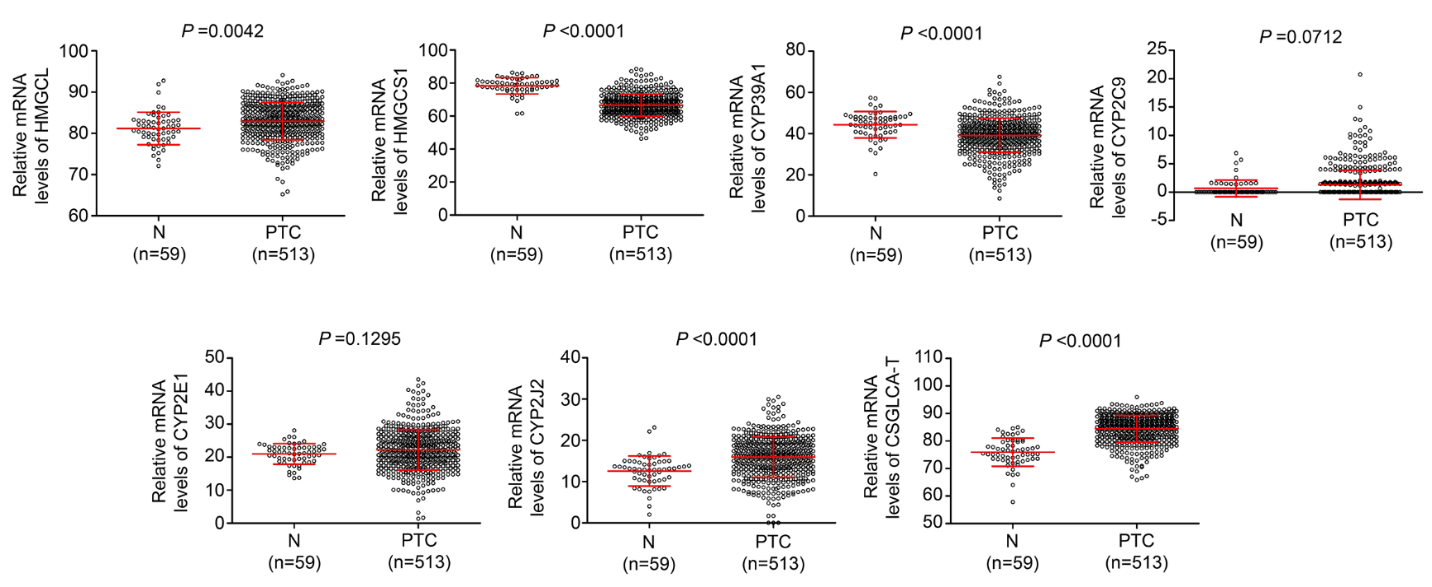
**

Figure. S1. Increased expression of HMGCL, HMGCS1, CYP39A1, CYP2C9, CYP2E1, CYP2J2 and CSGLCA-T in PTCs

mRNA expression of HMGCL, HMGCS1, CYP39A1, CYP2C9, CYP2E1, CYP2J2, CSGLCA-T were analyzed in PTCs and normal thyroid tissues (N) from TCGA database. Error bars represent SD.

**
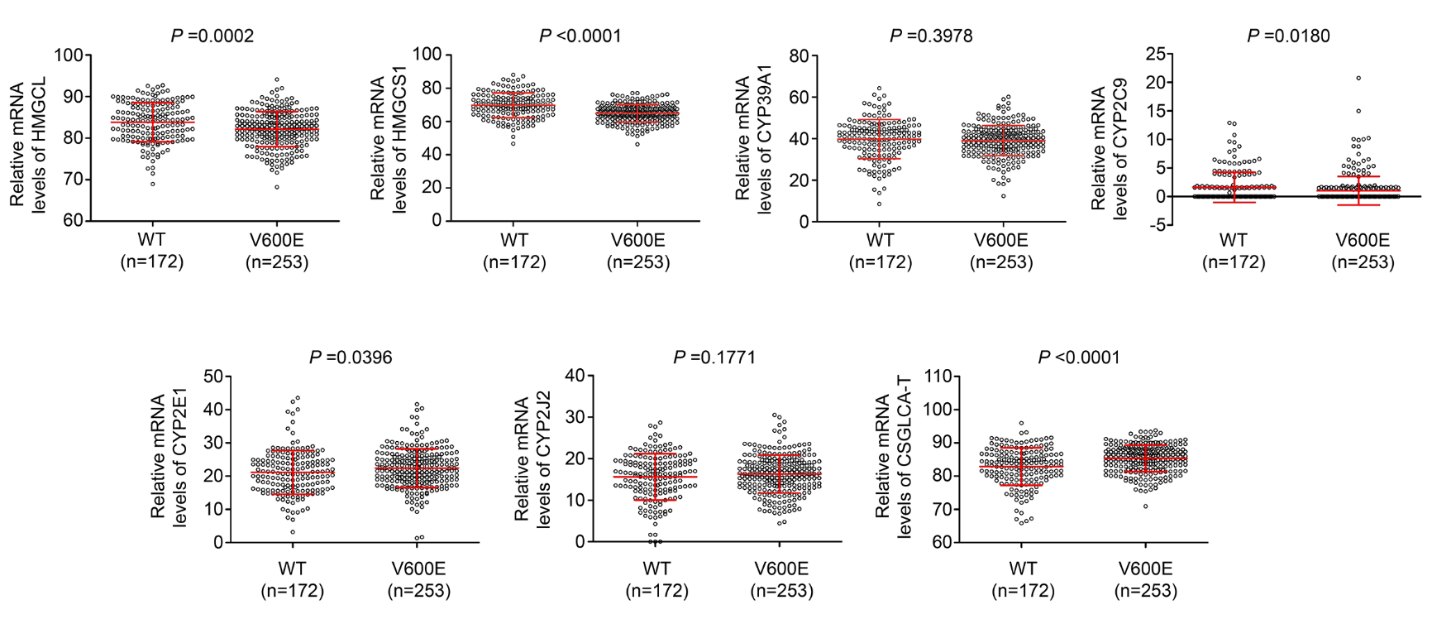
**

Figure. S2. Expression of 7 metabolism related genes in BRAF^V600E^ PTCs

mRNA expression of the 7 metabolism related genes in BRAF^V600E^ mutated (V600E) and BRAF wild-type (WT) PTCs from TCGA) database. Error bars represent SD.

**
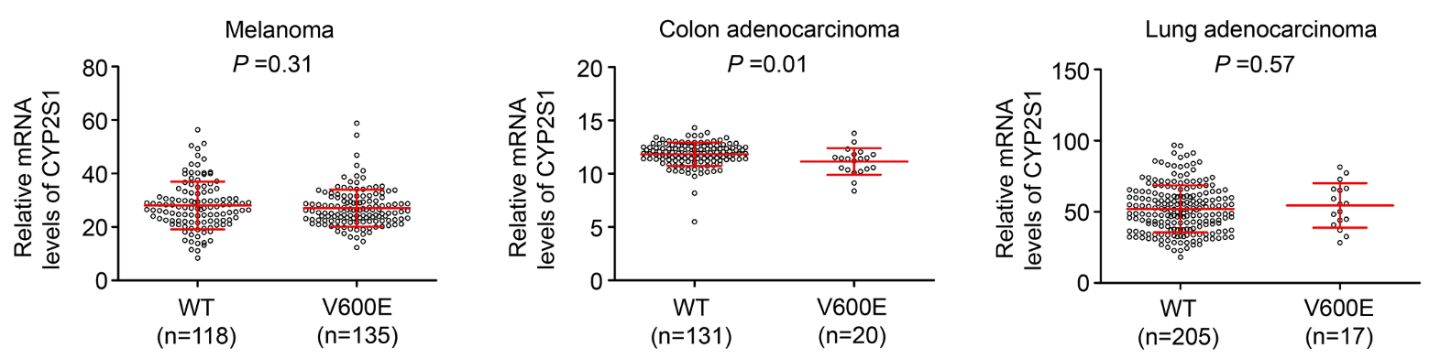
**

Figure. S3. Expression of CYP2S1 in different type of BRAF^V600E^ cancers

mRNA expression of CYP2S1 in BRAF^V600E^ mutated (V600E) and BRAF wild-type (WT) melanomas, colon adenocarcinomas and lung adenocarcinomas (data from TCGA database). Error bars represent SD.

**
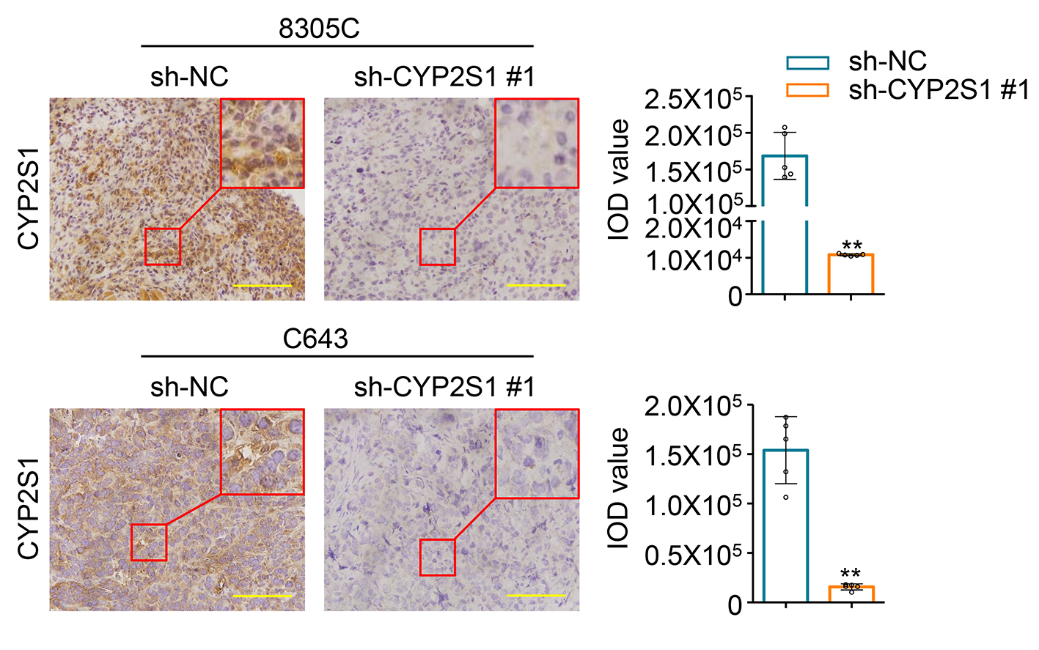
**

Figure. S4. Validation of CYP2S1 expression in the xenograft tumors

Validation of CYP2S1 expression in the indicated xenograft tumors (left panels). Scale bars, 200 µm. Statistical analysis was shown in right panels. Error bars represent SD. **, *P* <0.01.

**
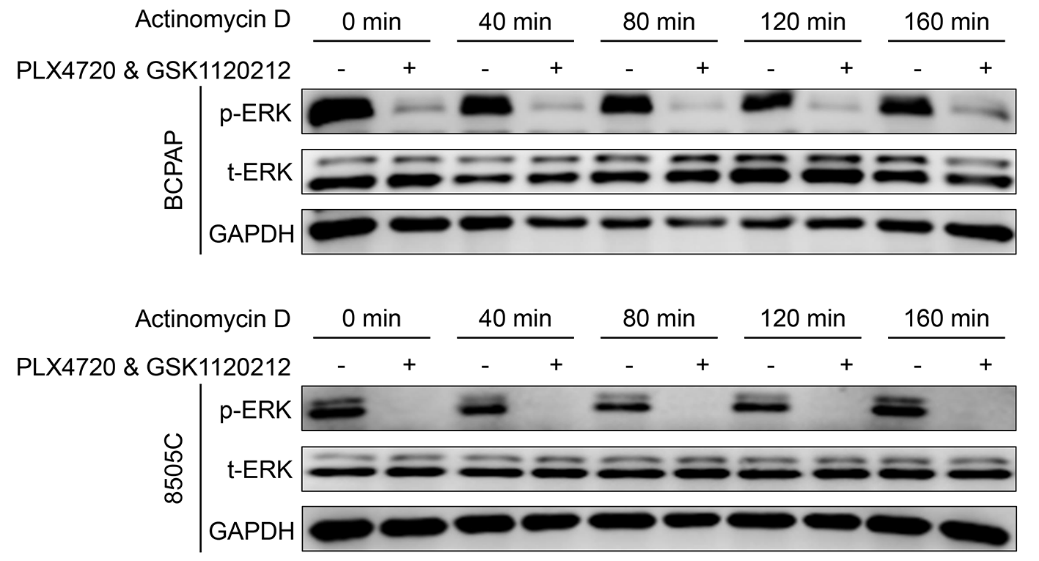
**

Figure. S5. Western blot to verify the inhibition of ERK phosphorylation

Western blot was performed to test the effect of the indicated treatments on ERK phosphorylation. GAPDH was used as a loading control.

**
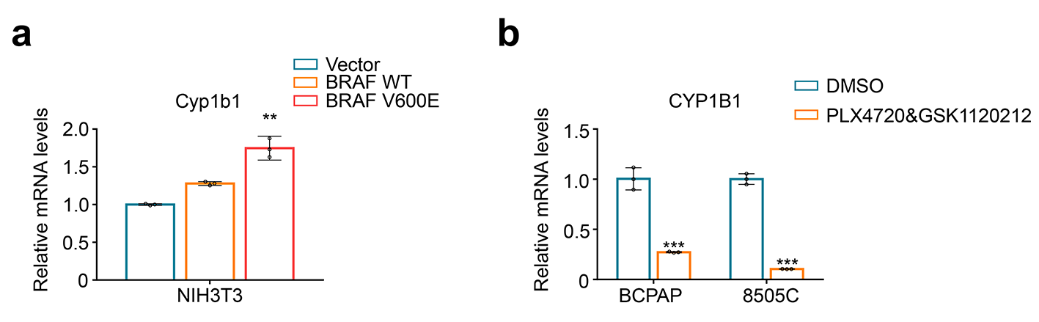
**

Figure. S6. Upregulation of CYP1B1 expression by BRAF^V600E^-mediated MAPK/ERK cascade

**a.** qRT-PCR was performed to evaluate the effect of ectopic expression of wild-type BRAF (BRAF WT) and BRAF^V600E^ on the expression of a downstream target of Ahr, Cyp1b1, in NIH3T3 cells. **b.** BCPAP and 8505c cells were treated with a combination of 1 μM PLX4720 and 500 nM GSK1120212 for 24 h, and qRT-PCR was performed to evaluate its effect on CYP1B1 expression. 18S rRNA was used as a reference gene. Error bars represent SD. **, *P* <0.01; ***, *P* <0.001.


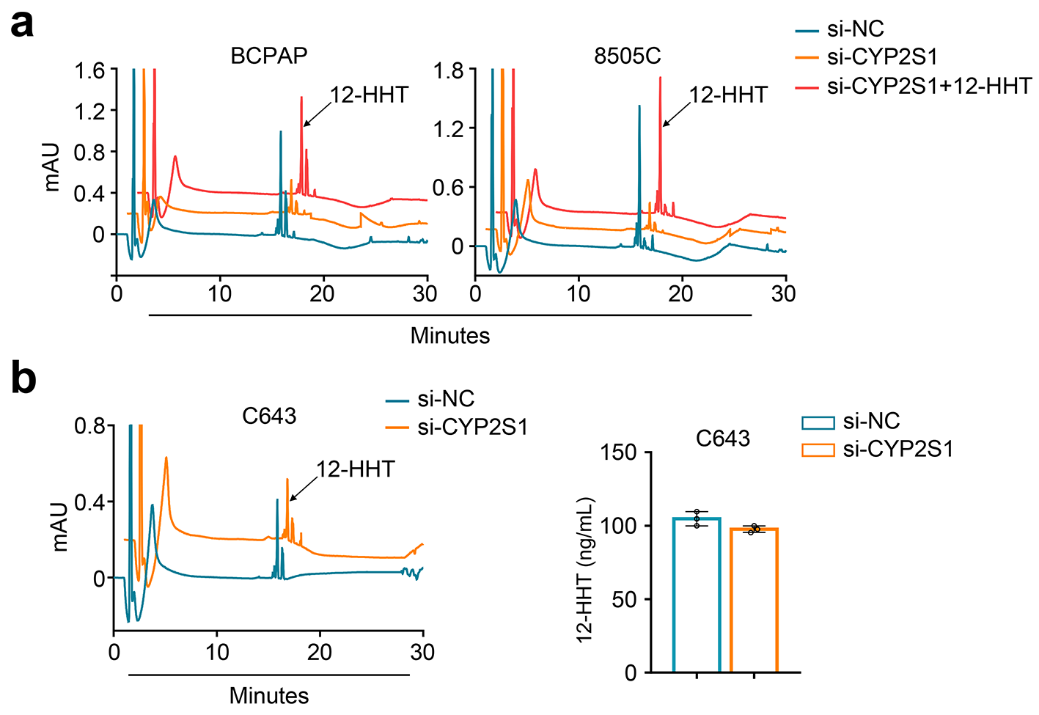


Figure. S7. Quantitative analysis of 12-HHT levels in thyroid cancer cells

**a.** HPLC was employed to analyze 12-HHT levels in BCPAP and 8505C cells. **b.** HPLC was employed to analyze 12-HHT levels in C643 cells. Representative images were shown in left panel. Quantitative analysis was shown in right panel. Error bars represent SD.


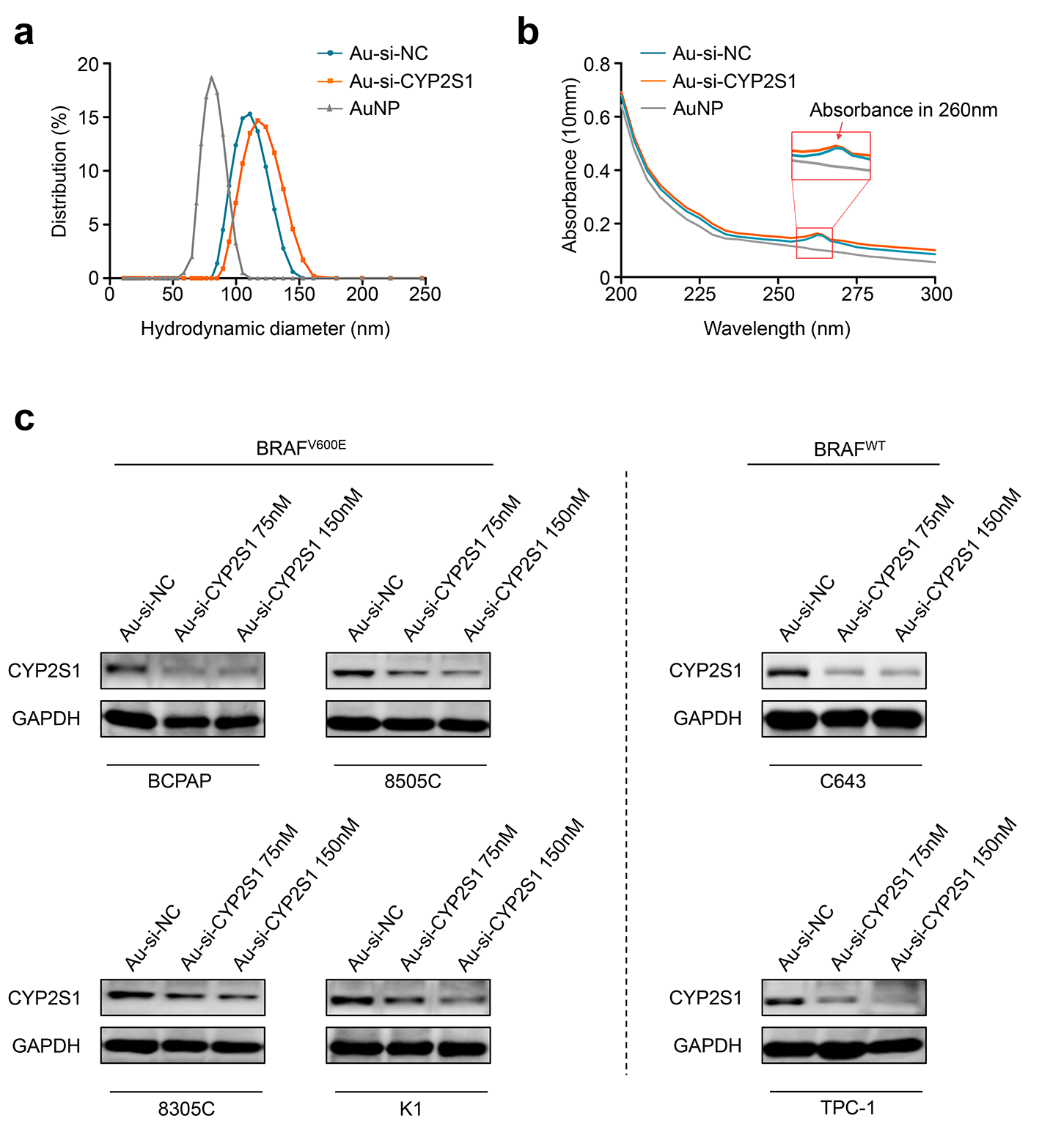


Figure. S8. Validation of the characters and function of Au-si-RNAs

Hydrodynamic distributions (**a**) and ultraviolet-visible spectra (**b**) of Au-si-RNAs and Au core (Au-NP) were measured in PBS buffer. Arrow indicates absorbance in 260 nm. **c.** BCPAP, 8305C, 8505C, K1, C643 and TPC-1 cells were treated with Au-si-NC and the indicated concentrations of Au-si-CYP2S1, and western blot analysis was then performed to validate inhibition of CYP2S1 expression. GAPDH was used as a loading control.


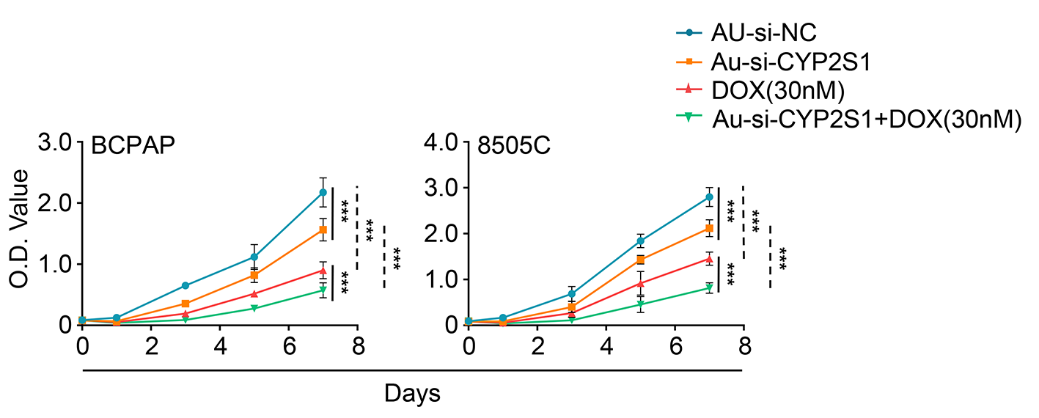


Figure. S9. Au-si-CYP2S1 enhanced the antitumor activities of DOX

The effect of the indicated treatments on the proliferation of BCPAP and 8505C cells were determined by MTT assay. Error bars represent SD. ***, *P* <0.001.


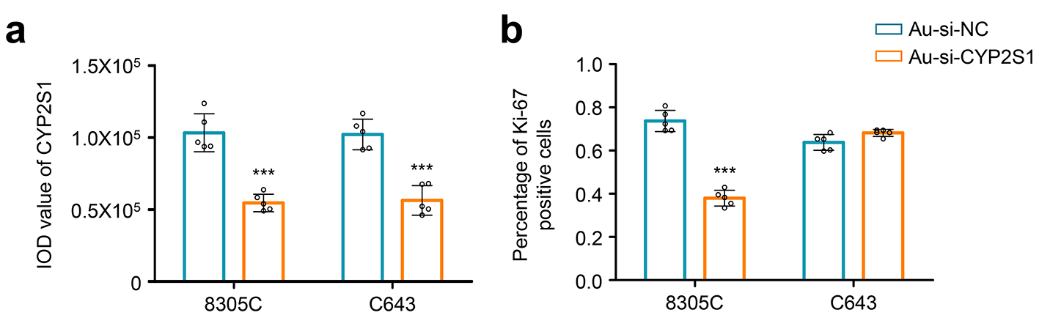


Figure. S10. Statistical analysis of CYP2S1 expression and the percentage of Ki-67 positive cells in the xenograft model

Statistical analysis of CYP2S1 expression (a) and the percentage of Ki-67 positive cells (b) in the indicated xenograft tumors. Error bars represent SD. ***, *P* <0.001.


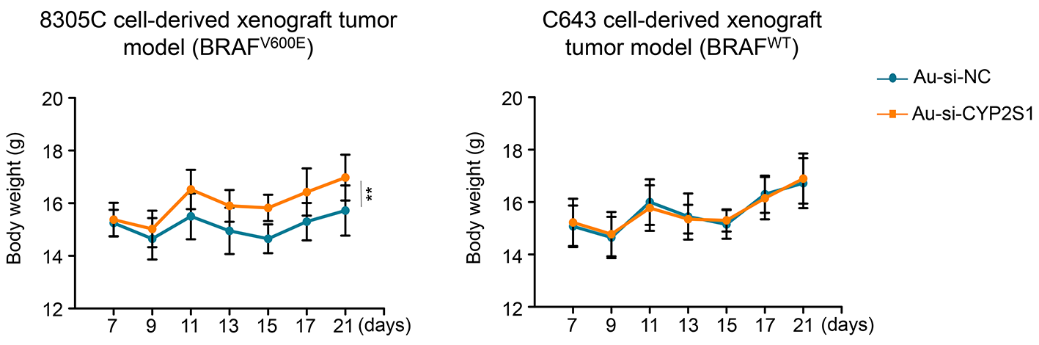


Figure. S11. The body weight of mice

Growth curves of body weight of mice with the indicated treatments. Left panel shows the 8305C cell-derived xenograft tumor model; Right panel shows the C643 cell-derived xenograft tumor model. Error bars represent SD. **, *P* <0.01.


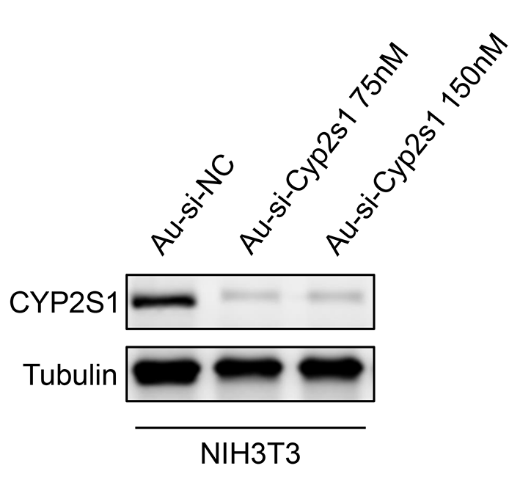


Figure. S12. Validation of the inhibition of Cyp2s1 expression in NIH3T3 cell

Western blot analysis was performed to validate inhibition of Cyp2s1 expression in NIH3T3 cells with the indicated treatments. Tubulin was used as a loading control.

**
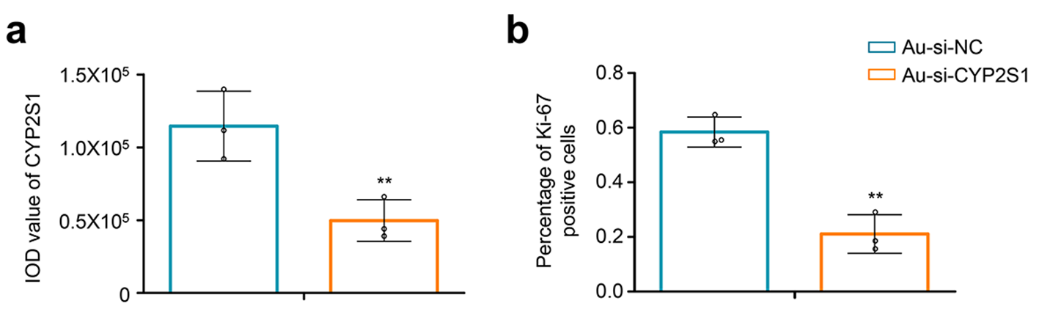
**

Figure. S13. Statistical analysis of Cyp2s1 expression and the percentage of Ki-67 positive cells in the transgenic model

Statistical analysis of Cyp2s1 expression (**a**) and the percentage of Ki-67 positive cells (**b**) in the indicated tumor tissues. Error bars represent SD. **, *P* <0.01.


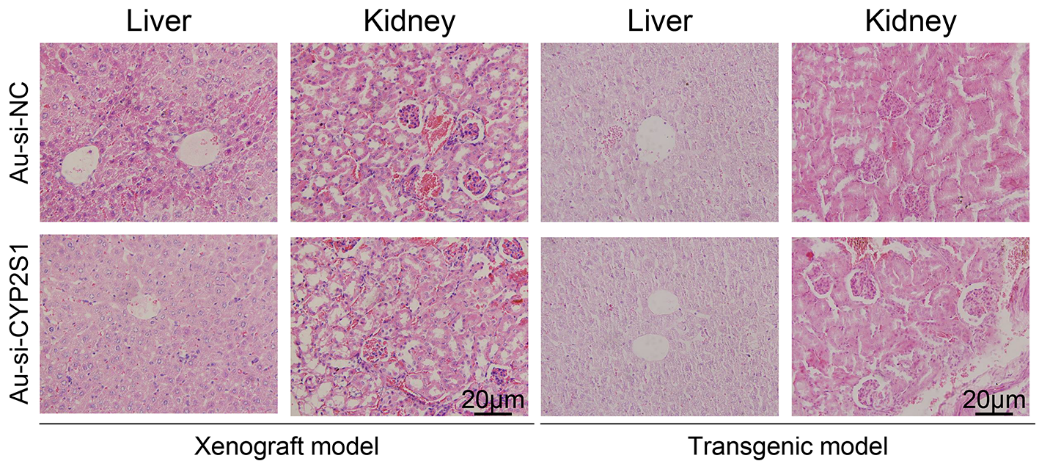


Figure. S14. H&E-stained of liver and kidney

The representative images of H&E-stained liver and kidney sections in the mice with the indicated treatments.

Table S1. siRNAs and shRNAs used in this study

| **siRNAs** | **Sequence (5’-3’)** |
| --- | --- |
| si-NC | UUCUCCGAACGUGUCACGUTT |
| si-CYP2S1 #1 | CAGCUGAGGAAGUUUACCATT |
| si-CYP2S1 #2 | CCUGAUGAAAUACCCUCAUTT |
| si-AHR #1 | GCUCUGAAUGGCUUUGUAUTT |
| si-AHR #2 | GCAGCUGAUAUGCUUUAUUTT |
| si-Ahr #1 (mouse) | CCUCCACUAUCCAAGAUUATT |
| sh-NC | GATCCGTTCTCCGAACGTGTCACGTAATTCAAGAGATTACGTGACACGTTCGGAGAATTTTTTC |
| sh-CYP2S1 | GATCCGCAGCTGAGGAAGTTTACCATTCAAGAGATGGTAAACTTCCTCAGCTGTTTTTTC |
| Thiolated si-CYP2S1 | CAGCUGAGGAAGUUUACCATT |
| Thiolated si-CYP2S1(mouse) | CGTTTGCCCTATGACGATAAA |

Table S2. The primers used in this study for plasmid construction

| **Constructs** | **Forward primer (5’-3’)** | **Reverse primer (5’-3’)** | **Restriction site** |
| --- | --- | --- | --- |
| pcDNA3.1(−)A-AHR | CCGCTCGAGGCCACCATGAACAGCAGCAGCGCC | CCCAAGCTTCAGGAATCCACTGGATGTCAAATCA | *Xho I* & *Hind III* |

Table S3. Antibodies used in this study

| **Antibodies** | **Catalog#** | **Source** |
| --- | --- | --- |
| anti-AHR | Ab2769 | Abcam |
| anti-GAPDH | AP0063 | Bioworld Technology |
| anti-Ki67 | 550609 | BD Pharmingen |
| anti-ERK1/2 | #4695 | Cell Signaling Technology |
| anti-p-ERK1/2 | #4370 | Cell Signaling Technology |
| anti-CYP2S1 | Sc-365806 | Santa Cruz |
| anti-BRAF | Sc-9002 | Santa Cruz |
| anti-CYP2S1 (mouse) | Sc-515464 | Santa Cruz |
| anti-β-Tublin | Sc-9104 | Santa Cruz |

Table S4. qRT-PCR primers used in this study

| **Genes** | **Forward primer (5’-3’)** | **Reverse primer (5’-3’)** |
| --- | --- | --- |
| Human CYP2S1 | GATGCTGGAAGGGACTTTTG | GATCAGCTCCTCGCCTTCT |
| Human AHR | TCAGTTCTTAGGCTCAGCGTC | AGTTATCCTGGCCTCCGTTT |
| Human CYP1A1 | CCCAGCTCAGCTCAGTACCT | GAGGCCAGAAGAAACTCCGT |
| Human CYP1B1 | TATCACTGACATCTTCGGCG | ACCTGATCCAATTCTGCCTG |
| Mouse Cyp2s1 | AAGAGAAGCCTTGGGAGGTC | GTAGAGCGAGCAGGGTGAAT |
| Mouse Ahr | TAGGCTCAGCGTCAGCTACC | CTCCTTCTTGCAAATCCTGC |
| Mouse Cyp1a1 | TTCTTTTGGGAGGAAGTGGA | ATCCAAGGCAGAATACGGTG |
| Mouse Cyp1b1 | AGCCAGGACACCCTTTCC | CACAACCTGGTCCAACTCAG |
| 18S | CGCCGCTAGAGGTGAAATTC | CTTTCGCTCTGGTCCGTCTT |

Table S5. The primers used in this study for luciferase reporter plasmid construction

| **Constructs** | **Position** | **Forward primer (5’-3’)** | **Reverse primer (5’-3’)** | **Restriction sites** |
| --- | --- | --- | --- | --- |
| pGL3-CYP2S1-Luc | Promoter  (-1562/+31) | ccgCTCGAGTGGGAGTGAAGTGTACTGGT | cccAAGCTTTGATGTCGGGAACTCATGCT | *Xho I* & *Hind III* |
| pGL3-CYP1B1-Luc | Promoter  (-1631/+80) | CATTTCTCTATCGATAGGTACCCTAGCCCCACGTGCATTTT | CAGTACCGGAATGCCAAGCTTCTCACAACTGGAGTCGCAGA | *Kpn I* & *Hind III* |
